# Supplementary material for: Mapping of QTLs Associated with Yield and Yield Related Traits in Durum Wheat (Triticum durum Desf.) Under Irrigated and Drought Conditions
Source: Int J Mol Sci. 2020 Mar 30;21(7):2372. doi: 10.3390/ijms21072372 (PMC7177892; doi:10.3390/ijms21072372)
Supplement: Supplementary file 1 [file ijms-21-02372-s001.pdf]

Article title: Mapping of QTLs associated with yield and yield related traits in durum wheat (*Triticum durum* Desf.) under irrigated and drought conditions
Journal Name: International Journal of Molecular Sciences

Mian Abdur Rehman Arif[1], Fauzia Attaria[1], Sajid Shokat[1], Saba Akram[1], Muhammad Qandeel Waheed[1], Anjuman Arif[1], Andreas Börner[2]*

[1]Nuclear Institute for Agriculture and Biology, Jhang Road, Faisalabad, Pakistan
[2]Leibniz Institute of Plant Genetics and Crop Plant Research, Corrensstr. 3, 06466 Seeland OT, Gatersleben, Germany
*Corresponding author (Email, m.a.rehman.arif@gmail.com)

Table S1 Correlations among Gr, Hd and Ph under various conditions and seasons, *: correlation significant at *p value* 0.05 and **: correlation significant at *p value* 0.01

	**Gr_S1**	**GrD_S1**	**Gr_S2**	**GrD_S2**	**Hd_S1**	**HdD_S1**	**Hd_S2**	**HdD_S2**	**Ph_S1**	**PhD_S1**	**Ph_S2**	**PhD_S2**
**Gr_S1**	1											
**GrD_S1**	0.63**	1										
**Gr_S2**			1									
**GrD_S2**		0.21**	0.44	1								
**TT_S1**												
**TT_S2**												
**Hd_S1**	0.23**	0.21**			1							
**HdD_S1**					0.94**	1						
**Hd_S2**		0.21**			0.8**	0.78**	1					
**HdD_S2**			0.21*		0.73**	0.78**	0.9**	1				
**Ph_S1**		-0.3**			-0.23**			-0.21**	1			
**PhD_S1**	-0.21**	-0.19*		0.24*					0.8**	1		
**Ph_S2**		-0.19*						-0.2*	0.73**	0.68**	1	
**PhD_S2**									0.71**	0.65**	0.89**	1

Table S2 Correlations among Tr, Sc, Phr and Wue under various conditions and seasons, *: correlation significant at *p value* 0.05 and **: correlation significant at *p value* 0.01

	Tr_S1	TrD_S1	Tr_S2	TrD_S2	Sc_S1	ScD_S1	Sc_S2	ScD_S2	Phr_S1	PhrD_S1	Phr_S2	PhrD_S2	Wue_S1	WueD_S1	Wue_S2	WueD_S2
**Tr_S1**	1															
**TrD_S1**	0.21**	1														
**Tr_S2**	0.54**	-0.36**	1													
**TrD_S2**			0.25**	1												
**Sc_S1**	0.5**		0.27**		1											
**ScD_S1**	0.2*	0.85**	-0.39**			1										
**Sc_S2**				-0.28**	0.38**		1									
**ScD_S2**		0.54**	-0.39**		-0.21**	0.66**	0.47**	1								
**Phr_S1**					0.84**		0.35**		1							
**PhrD_S1**						0.36**		0.26**		1						
**Phr_S2**	-0.23**	0.25**	-0.53**	-0.38**		0.33**	0.79**	0.61**	0.28**	0.21**	1					
**PhrD_S2**		0.48**	-0.44**	-0.4**		0.61**	0.5**	0.96**		0.3**	0.68**	1				
**Wue_S1**	-0.29**		-0.52**	-0.21**	0.56**		0.27**		0.83**		0.5**		1			
**WueD_S1**		-0.75**				-0.4**		-0.27**		0.65**		-0.21**		1		
**Wue_S2**	-0.37**	0.3**	-0.75**	-0.37**		0.37**	0.52**	0.57**			0.93**	0.65**	0.53**		1	
**WueD_S2**		0.39**	-0.44**	-0.58**		0.54**	0.5**	0.86**		0.33**	0.69**	0.97**			0.67**	1

Table S3 Correlations among Sl, Sw, Sps and Tkw under various conditions and seasons, *: correlation significant at *p value* 0.05 and **: correlation significant at *p value* 0.01

	**Sl_S1**	**SlD_S1**	**Sl_S2**	**SlD_S2**	**Sw_S1**	**SwD_S1**	**Sw_S2**	**SwD_S2**	**Sps_S1**	**SpsD_S1**	**Sps_S2**	**SpsD_S2**	**Tkw_S1**	**TkwD_S1**	**Tkw_S2**	**TkwD_S2**
**Sl_S1**	1															
**SlD_S1**	0.47**	1														
**Sl_S2**		0.3**	1													
**SlD_S2**		0.26**	0.67**	1												
**Sw_S1**	0.25**				1											
**SwD_S1**	0.27**	0.33**			0.6**	1										
**Sw_S2**	0.27**		0.25**				1									
**SwD_S2**				0.22**		0.27**	0.57**	1								
**Sps_S1**					0.68**	0.57**			1							
**SpsD_S1**	0.29**	0.3**			0.54**	0.9**		0.28**	0.63**	1						
**Sps_S2**							0.57**	0.37**			1					
**SpsD_S2**				0.23**			0.34**	0.74**			0.36**	1				
**Tkw_S1**				-0.22**	0.36**	0.25**	0.22**	0.29**	0.26**				1			
**TkwD_S1**				0.21**	0.26**	0.3**	0.23**	0.31**	0.22**				0.8**	1		
**Tkw_S2**				-0.22**	0.25**	0.25**	0.58**	0.66**	0.24**		0.2*	0.29**	0.41	0.36**	1	
**TkwD_S2**					0.21**	0.23**	0.53**	0.73**	0.26**			0.31**	0.43**	0.47**	0.9**	1

Table S4 Correlations among Bm, Yd and HI under various conditions and seasons, *: correlation significant at *p value* 0.05 and **: correlation significant at *p value* 0.01

	**Bm_S1**	**BmD_S1**	**Bm_S2**	**BmD_S2**	**Yd_S1**	**YdD_S1**	**Yd_S2**	**YdD_S2**	**HI_S1**	**HID_S1**	**HI_S2**	**HID_S2**
**Bm_S1**	1											
**BmD_S1**	0.44**	1										
**Bm_S2**	0.58**		1									
**BmD_S2**		0.22**	0.32**	1								
**Yd_S1**	0.63**	0.3**	0.33**		1							
**YdD_S1**	0.29**	0.69**			0.49**	1						
**Yd_S2**	0.43**		0.74**	0.32**	0.63**		1					
**YdD_S2**		0.32**	0.25**	0.74**	0.3**	0.5**	0.44**	1				
**HI_S1**	-0.2*		-0.28**		0.5**	0.35**			1			
**HID_S1**	-0.24**	-0.38**	-0.2*			0.34**		0.22*	0.4**	1		
**HI_S2**					0.46**		0.5**	0.32**	0.64**	0.44**	1	
**HID_S2**				-0.3**	0.24**	0.49**		0.37**	0.21**	0.44**	0.3**	1

Table S5 Correlations among STI, MP, STI, SSI and DRI in two seasons, *: correlation significant at *p value* 0.05 and **: correlation significant at *p value* 0.01

	**STI_S1**	**STI_S2**	**MP_S1**	**MP_S2**	**ST_S1**	**ST_S2**	**SS1_S1**	**SSI_S2**	**DRI_S1**	**DRI_S2**
**STI_S1**	1									
**STI_S2**	0.55**	1								
**MP_S1**	0.95**	0.56**	1							
**MP_S2**	0.51**	0.91**	0.59**	1						
**ST_S1**	0.67**	0.5**	0.87**	0.63**	1					
**ST_S2**	0.31**	0.54**	0.49**	0.83**	0.68**	1				
**SSI_S1**	0.2*	0.31**	0.45*	0.54**	0.8**	0.75**	1			
**SSI_S2**			0.26*	0.34**	0.54**	0.78**	0.76**	1		
**DRI_S1**	0.31**			-0.21*	-0.4**	-0.49**	-0.81**	-0.63**	1	
**DRI_S2**	0.27*	0.55**		0.26**		-0.32**	-0.39**	-0.77**	0.48**	1

Table S6 Rainfall data of the two seasons of the study

	2016-17 (S1)		2017-18 (S2)	
	**Amount in mm**	**Days**	**Amount in mm**	**Days**
**Nov**	0	0	0.44	0
**Dec**	0	0	0.06	0
**Jan**	11.14	5	0	0
**Feb**	1.22	1	7.25	3
**March**	4.71	2	10.56	7
**April**	16.58	4	12.21	15
**May**	2.6	2	11.96	5
**Total**	36.25	14	42.48	30

Table S7 Temperature data of the two seasons of the study in °C

	2016-17 (S1)			2017-18 (S2)		
	**Min.**	**Max.**	**Mean**	**Min.**	**Max.**	**Mean**
Nov	16	31	22	17	19	22
Dec	12	27	18	13	24	17
Jan	9	21	13	10	25	15
Feb	11	27	18	13	26	18
March	14	31	22	17	32	24
April	23	39	31	23	36	29
May	29	42	36	30	41	36
**Mean**	16.28	31.14	22.85	17.57	29	23

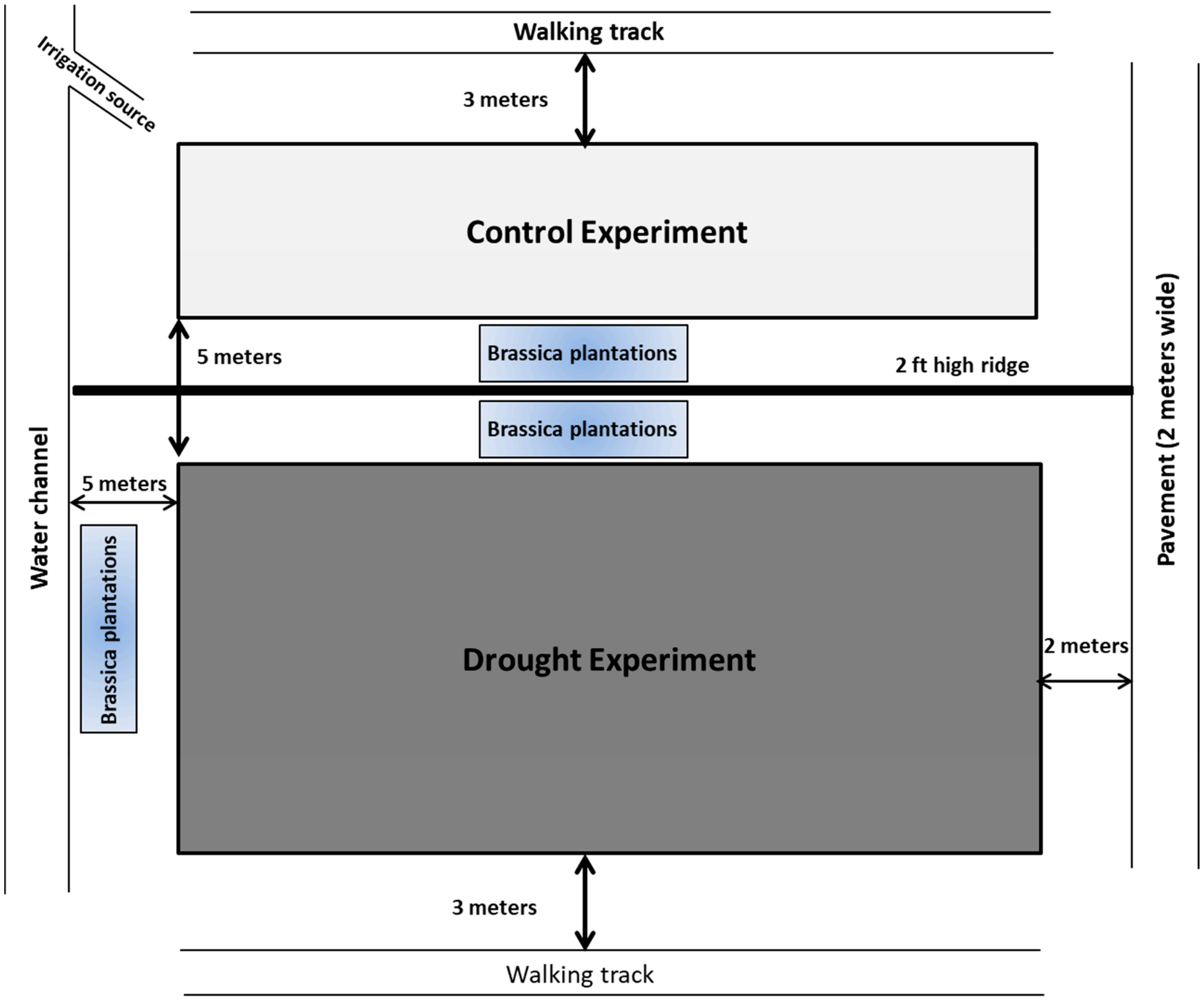

Fig S1 Layout of the experiments in S1 and S2
